# TB treatment using family members, treatment supporters and self-administered therapies in rural Papua New Guinea

**DOI:** 10.5588/pha.22.0062

**Published:** 2023-06-21

**Authors:** G. Kurbaniyazova, F. Msibi, H. Bogati, M. Kal, A. Sofa, E. Abdi Djama, P. Mozi, F. Hossain, P. Blasco, L. Sannino

**Affiliations:** 1 Médecins San Frontières (MSF), Paris, France; 2 National Tuberculosis Programme, Papua New Guinea

**Keywords:** DOT, DS-TB, PNG, SAT, alternative TB treatment delivery

## Abstract

**SETTING:**

Papua New Guinea (PNG) has one of the world’s highest TB incidence rates. It is difficult for patients to access TB care in remote provinces due to insufficient infrastructure and challenging terrain, making varied, targeted delivery models for treating TB necessary.

**OBJECTIVE:**

To assess treatment outcomes using self-administered treatment (SAT), family-supported treatment and community-based directly observed therapy (DOT) via treatment supporter (TS) in the PNG context.

**DESIGN:**

A retrospective, descriptive analysis of routinely collected data from 360 patients at two sites in 2019–2020. All patients were assigned a treatment model based on risk factors (adherence or default) and offered patient education and counselling (PEC), family counselling and transportation fees. End-of-treatment outcomes were assessed for each model.

**RESULTS:**

Treatment success rates among drug-susceptible TB (DS-TB) were good overall: 91.1% for SAT, 81.4% for family-supported treatment and 77% for DOT patients. SAT was strongly associated with favourable outcomes (OR 5.7, 95% CI 1.7–19.3), as were PEC sessions (OR 4.3, 95% CI 2.5–7.2).

**CONCLUSION:**

By considering risk factors when determining their treatment delivery model, strong outcomes were seen in all three groups. Multiple modes of treatment administration, tailored to individuals’ needs and risk factors, is a feasible, effective, patient-centred care model for hard-to-reach, resource-limited settings.

## Abstract (fr)

**LIEU:**

La Papouasie-Nouvelle-Guinée (PNG) présente l’un des taux d’incidence de TB les plus élevés au monde. Il est difficile pour les patients d’accéder aux soins contre la TB dans les provinces éloignées en raison d’une infrastructure insuffisante et d’un terrain difficile, rendant nécessaire l’utilisation de modèles de prestation variés et ciblés pour le traitement de la tuberculose.

**OBJECTIF:**

Évaluer les résultats du traitement en utilisant l’autotraitement, le traitement soutenu par la famille et la thérapie directement observée (DOT) basée sur la communauté avec un accompagnateur de traitement, dans le contexte de la PNG.

**MÉTHODES:**

Une analyse rétrospective et descriptive des données collectées de manière routinière auprès de 360 patients dans deux sites entre 2019 et 2020. Tous les patients ont été assignés à un modèle de traitement en fonction des facteurs de risque (adhésion ou abandon) et ont bénéficié d’une éducation et d’un accompagnement des patients, de conseils aux familles et de frais de transport. Les résultats en fin de traitement ont été évalués pour chaque modèle.

**RÉSULTATS:**

Les taux de réussite du traitement de la TB sensible aux médicaments (DS-TB) étaient globalement bons: 91,1% pour l’autotraitement, 81,4% pour le traitement soutenu par la famille et 77% pour les patients en DOT. L’autotraitement était fortement associé à des résultats favorables (OR 5,7; IC 95% 1,7–19,3), tout comme les séances d’éducation et d’accompagnement des patients (OR 4,3; IC 95% 2,5–7,2).

**CONCLUSION:**

En tenant compte des facteurs de risque lors de la détermination du modèle de prestation de traitement, de bons résultats ont été observés dans les trois groupes. Plusieurs modes d’administration du traitement, adaptés aux besoins et aux facteurs de risque individuels, constituent un modèle de soins réalisable, efficace et centré sur le patient pour les contextes difficiles d’accès et aux ressources limitées.

TB and drug-resistant TB (DR-TB) pose a significant public health threat, especially in high-burden countries such as Papua New Guinea (PNG).^1^ In PNG, extreme geography, poor road access, minimal healthcare infrastructure and excessively high transportation costs combine to prevent many TB patients from accessing healthcare services. Daily visits to a healthcare provider for directly observed TB therapy (DOT) can be particularly burdensome and contribute to high dropout rates from treatment.

PNG’s Gulf Province is especially remote and dominated by mountains, lowland river deltas and grassland flood plains that are difficult to navigate in areas without extensive road networks. The region also has one of the highest TB burdens in the country (564 cases/100,000 population) and poor treatment outcomes (only 73% of bacteriologically confirmed TB was successfully treated from 2008 to 2016).^2^ To address the high default rates in this neglected, rural and isolated area of PNG, a partnership between Médecins Sans Frontières (MSF) and the PNG Ministry of Health (MoH) began offering alternative methods of TB treatment delivery to people diagnosed with drug-susceptible TB (DS-TB) in 2019. These methods included self-administered treatment (SAT) and family therapy models (FTMs), in addition to the recommended model of community-based directly observed therapy under the care of a non-clinical treatment supporter (DOT-TS).^3^

DOT alternatives have been used in other rural, remote and even conflict-affected settings globally where daily, facility-based DOT is difficult or impossible to deliver,^4–7^ as well as in other contexts,^8,9^ with successful results. All evidence points to community DOT as a feasible alternative to facility-based DOT; however, definite conclusions for non-DOT options cannot be drawn yet, as systematic reviews are contradictory, some finding no significant difference between the models,^10^ while others show improved results for DOT.^11^ It is therefore necessary to accumulate more evidence from diverse settings, given that facility-based DOT is often not an option in remote areas with dispersed populations (like Gulf Province), may sometimes pose ethical challenges for health workers and can be a significant strain for patients.^12–14^ Our study adds to the evidence base surrounding community- and home-based DOT alternatives, describing end-of-treatment outcomes in a cohort receiving treatment for DS-TB by using alternative TB treatment models that did not rely on health facility-based DOT.

## STUDY POPULATION, DESIGN AND METHODS

Using routinely collected patient data, we retrospectively analysed the outcomes of DS-TB treatment under three modes of administration (MoA) (SAT, FTM, DOT-TS). Patients aged 14–60 years with bacteriologically confirmed TB, who started TB treatment from July 2019 to June 2020 under an alternative treatment model (SAT, FTM, DOT-TS) in the Kerema or Malalua Basic Management Units (BMUs) of Gulf Province were eligible for participation. Adherence was assessed at each patient encounter, whether for follow-up reviews at the clinic, medication refill appointments or home visitation. When adherence issues were identified, supportive counselling sessions were provided.

### Modes of administration

Patients were assigned to one of the following MoAs: self-administered treatment (SAT), patients were given monthly drug supplies to be taken at home without supervision; family therapy model (FTM), patients were given monthly drug supplies to be taken at home with the supervision of a family member previously trained at the clinic; directly observed treatment (DOT) by treatment supporters (TS), patients were assigned a trained TS who was responsible of directly observing the patient while taking his/her daily medications. MoAs were assigned at treatment initiation. Eligibility for the SAT model was considered for patients 14–60 years of age with satisfactory TB knowledge and no physical or mental disabilities. Adherence was assessed when they came for clinical review or drug refills. FTM was assigned to patients not eligible for SAT who were able to visit a participating health facility with their family TS for an eligibility assessment. This family member was also required to attend a DOT training course, have satisfactory TB knowledge and no physical or mental disabilities. Family members needed to live at a reasonable distance from the patient’s home (and have no plans to move or be away during the treatment period), have the capacity to recognise possible side effects and adherence problems (and inform the medical team in a timely manner), fill out the patient’s treatment card and independently provide DOT. Finally, trained community TSs were assigned to individuals who were not eligible for either SAT or FTM or had a history of TB treatment interruption. These patients received community-based DOT either at home or in a place previously arranged with the TS. In some cases, patients were also assigned to the DOT-TS group if there were other concerns or when it was advised by a clinician. All patients remained under the MoA that they were assigned at initiation throughout their treatment.

All participants were counselled by a patient education and counselling (PEC) team member prior to being assigned a mode of administration. DS-TB patients underwent three mandatory PEC sessions: at treatment initiation, after 2 weeks and when they transitioned to a new phase of treatment at 2 months. Patients received a monthly supply of TB medicines at their nearest BMU, were monitored for drug tolerance for the first 2 weeks of treatment and were seen by clinicians when side effects or adherence issues occurred, a new treatment phase began, follow-up sputum tests were conducted or a treatment outcome was determined.

### Statistical analysis

All demographic and clinical data were entered into a TB register and analysed using Stata v.16.1 (Stata, College Station, TX, USA). Descriptive analyses included medians with interquartile ranges (IQRs), means with their standard deviations (SDs) or counts with proportions. Proportions are provided with 95% confidence intervals (95% CIs) and P-values. Univariate analysis was performed using the following variables: treatment outcomes, MoA (SAT, FTM and DOT-TS), age, sex, marital status, treatment history and number of PEC sessions. We defined outcomes according to WHO guidelines.^15^ All statistical tests were computed as two-sided at α 0.05.

## RESULTS

### Study population

A total of 360 patients (Kerema BMU: 192; Malalaua BMU: 168) being treated for bacteriologically confirmed pulmonary and extrapulmonary DS-TB were included (DOT: 26; SAT: 237; FTM: 97). Four patients without a final treatment outcome recorded in the patient file were excluded. Baseline characteristics are shown in Table 1, while Tables 2 and 3 give factors associated with various treatment outcomes. Most patients (88%) had no previous TB treatment history.

**TABLE 1:** Baseline characteristics (*n* = 360) of drug-susceptible TB patients registered for treatment using alternative treatment delivery models in Gulf Province, Papua New Guinea, July 2019–June 2020

	Mode of administration			
	Treatment-supporter, directly observed (*n* = 26) *n* (%)	Self-administered (*n* = 237) *n* (%)	Family model (*n* = 97) *n* (%)	*P*-value
TB site				
Pulmonary	15 (58)	135 (57)	49 (51)	
Extrapulmonary	11 (42)	102 (43)	48 (49)	0.542
Age, years, mean ± SD	29 ± 12.9	32 ± 11.9	23 ± 17.4	0.000
Sex				
Male	13 (50)	113 (48)	45 (46)	
Female	13 (50)	124 (52)	52 (54)	0.944
Marital status				
Single	15 (58)	74 (31)	59 (61)	
Married	9 (34)	158 (67)	36 (37)	
Widow	1 (4)	4 (1.6)	2 (2)	
Divorced	1 (4)	1 (0.4)	0 (0)	0.000
Treatment history*				
New	10 (40)	217 (96)	89 (93)	
Previously treated	15 (60)	9 (4)	7 (7)	0.000
PEC sessions				
One	0	9 (4)	5 (5)	
Two	6 (23)	25 (10)	13 (14)	
Three	20 (77)	203 (86)	75 (81)	0.032

*Transferred-in patients excluded.

SD = standard deviation; PEC = patient education and counselling.

**TABLE 2:** Treatment outcomes of (*n* = 360) of drug-susceptible TB patients registered for treatment using alternative treatment delivery models in Gulf Province, Papua New Guinea, July 2019–June 2020

Variables	Outcome		*P*-value
	Unfavourable *n* (%)	Favourable *n* (%)	
Mode of administration			
Treatment-supporter, directly observed	6 (23)	20 (77)	
Self-administered	21 (9)	216 (91)	0.009
Family-model	18 (19)	79 (81)	
TB site			
Pulmonary	22 (11)	177 (89)	0.423
Extrapulmonary	23 (14)	138 (86)	
Age, years, mean ± SD	32.3 ± 16.3	29.5 ± 13.8	0.210
Sex			
Male	24 (14)	147 (86)	0.428
Female	21 (11)	168 (89)	
Marital status			
Single	29 (14)	174 (86)	0.244
Married	16 (10)	141 (90)	
Treatment history			
New	38 (12)	278 (88)	0.675
Previously treated	6 (19)	25 (81)	
PEC sessions, *n*, mean ± SD	2.2 ± 1.0	2.8 ± 0.4	0.001
Adherence	10 (3)	303 (97)	0.000
Sputum conversion	11 (7.7)	132 (92.3)	1.000

SD = standard deviation; PEC = patient education and counselling.

**TABLE 3:** Treatment outcomes by mode of administration of drug-susceptible TB patients registered for treatment using alternative treatment delivery models in Gulf Province, Papua New Guinea, July 2019–June 2020 (*n* = 360)

Treatment outcomes	Modes of administration			*P*-value
	Treatment-supporter, directly observed therapy *n* (%)	Self-administered therapy *n* (%)	Family model *n* (%)	
Favourable				
Cured	9 (35.0)	91 (38.4)	25 (25.8)	
Completed	11 (42.0)	125 (52.7)	54 (55.7)	
Unfavourable				
Died	1 (3.8)	6 (2.5)	13 (13.4)	<0.005
Failed	0 (0.0)	3 (1.3)	0 (0.0)	
Lost to follow-up	5 (19.2)	12 (5.1)	5 (5.2)	<0.005
Success rate, %	77.0	91.1	81.4	<0.005

Baseline characteristics varied widely between groups. The SAT group had a higher proportion of patients who had never received TB treatment before (96%), while 60% of patients under DOT had been previously treated for TB. Patients under SAT received significantly more PEC sessions (86% had three sessions vs. 77% of DOT patients) and had better adherence (99% of SAT patients had good adherence vs. 96% of DOT patients).

Treatment success rates were evaluated for each MoA group (Table 2). Overall success rates were good at 87.5%, while SAT showed a 91% success rate. FTM and DOT rates were respectively 81.4% and 77% (*P* < 0.005). Loss to follow-up (LTFU) was higher in patients treated using DOT (19.2%) than in the other groups (SAT: 5.1% vs. FTM: 5.2%; *P* < 0.05), and the mortality rate was higher in FTM patients (13.4%) than in the DOT-TS and SAT groups (3.8 vs. 2.5%, *P* < 0.05).

Across all the models, three people failed treatment (all from the SAT group), although no statistical significance was observed. No drug resistance data were available for these patients. Two of the failed cases were found to be adherent, while adherence for the third case was not recorded. All had no prior TB treatment history, had pulmonary TB and had been assigned a treatment duration of 6–8 months.

## DISCUSSION

This is the first descriptive analysis of alternative, community and home-based TB treatment models in PNG. Evidence describing alternatives to DOT in settings with poor road and health infrastructure are sparse, although many of the world’s TB patients have considerable barriers to reaching care and need more options for completing their TB treatment. Our findings confirm reports from parts of the world that community-based MoAs can achieve successful outcomes and could be considered for patients living in remote areas.^4–9^

Although the operational study design and small sample make it impossible to generalise these findings or make broad comparisons between the three MoAs, it is notable that by carefully considering a patient’s risk factors prior to determining how their treatment should be delivered, high rates of favourable outcomes were seen in the SAT group. These data show that having multiple treatment MoAs, tailored to individuals’ needs and risk factors, is a feasible and effective way to provide patient-centred care. In our cohort, DOT was reserved for those with prior adherence issues or other concerns, which likely contributed to the SAT group outperforming the other models in this analysis. The SAT group was also already more likely to have good knowledge of TB, were mostly treatment-naïve (while 60% of DOT patients had received TB treatment previously), had little LTFU among previously treated patients (2/9), and attended significantly more PEC sessions than DOT patients (*P* = 0.032). Lower attendance at PEC sessions in DOT group can be explained by early LTFU (thus missing the third session) and failure to show up at the clinic for adherence sessions (DOT patients were treated at home by TSs and home visits by a counsellor were not always possible). Prospective studies with larger samples are needed on this topic to definitively determine which of these variables is independently associated with better outcomes.

PEC had a clear and significant benefit for all patients, in line with previous research.^16–19^ Ongoing adherence education and counselling are crucial to empower patients to make informed decisions about their treatment and ensure their collaboration with health workers. This is especially important under SAT models. PEC is recommended in the National Guidelines,^3^ but standardised counselling sessions and dedicated counsellors might have been the added value for our project. While not enough to draw conclusions about the association between failure and SAT, the three cases of treatment failure in our SAT group indicate the importance of SAT adherence monitoring, possibly with digital adherence technologies.^20,21^

The high death rate on FTM probably reflected more severe disease in this group of patients, who were not able to take their drugs autonomously. Despite this, patients on FTM achieved 81% success rate with low LTFU (5.2%). This finding shows that this is also a valid MoA, in line with other studies.^10,22^ Family support in general is key to treatment success, as it can increase the patient’s confidence and positively affect adherence.^23^ Including family members in education and counselling can address the negative family perceptions of TB that ‘can make the treatment experience difficult’.^24^ In PNG, TB stigma can be extreme (a ‘shameful disease,’ an ‘HIV/AIDS-associated disease,’ or linked to ‘sorcery’ or ‘sinful practices’).^25^ Families exert considerable influence on healthcare decision-making and whether to involve traditional and homeopathic healing approaches.

LTFU also differed by MoA (*P* < 0.05, Table 3) and constituted 5.1% in SAT, 5.2% in FTM and 19.2% in DOT-TS patients. These outcomes suggest that the safety of the SAT model, as well as its association with favourable outcomes should be further investigated. Because of the small sample size and possible misclassification in each group, we are limited to descriptive analysis, and comparisons between the groups must be made with care. The limited treatment duration of 6–9 months, the once daily frequency of medication and the availability of fixed-dose combinations made it easier for people to self-administer their medication in ways that may not be able to be replicated with DR-TB treatment (further study is needed).

## CONCLUSION

This cohort demonstrated that SAT for DS-TB can be associated with high success rates in a resource-constrained setting with extreme geographical characteristics, especially when accompanied by strong PEC activities and the careful selection of patients. Expanding the model could bring fundamental changes for TB control, once further studies have helped us understand which factors are independently associated with positive outcomes and if the model is feasible for drug-resistant patients.
